# Transcriptomic investigation of wound healing and regeneration in the cnidarian *Calliactis polypus*

**DOI:** 10.1038/srep41458

**Published:** 2017-02-02

**Authors:** Zachary K. Stewart, Ana Pavasovic, Daniella H. Hock, Peter J. Prentis

**Affiliations:** 1 School of Earth, Environmental and Biological Sciences, Queensland University of Technology, 2 George Street, Brisbane, Australia; 2 School of Biomedical Sciences, Queensland University of Technology, 2 George Street, Brisbane, Australia; 3 Institute of Health and Biomedical Innovation, Queensland University of Technology, 2 George Street, Brisbane, Australia; 4 Biological Sciences Centre, Federal University of Santa Catarina, Florianópolis, Brazil; 5 Institute for Future Environments, Queensland University of Technology, 2 George Street, Brisbane, Australia.

## Abstract

Wound healing and regeneration in cnidarian species, a group that forms the sister phylum to Bilateria, remains poorly characterised despite the ability of many cnidarians to rapidly repair injuries, regenerate lost structures, or re-form whole organisms from small populations of somatic cells. Here we present results from a fully replicated RNA-Seq experiment to identify genes that are differentially expressed in the sea anemone *Calliactis polypus* following catastrophic injury. We find that a large-scale transcriptomic response is established in *C. polypus*, comprising an abundance of genes involved in tissue patterning, energy dynamics, immunity, cellular communication, and extracellular matrix remodelling. We also identified a substantial proportion of uncharacterised genes that were differentially expressed during regeneration, that appear to be restricted to cnidarians. Overall, our study serves to both identify the role that conserved genes play in eumetazoan wound healing and regeneration, as well as to highlight the lack of information regarding many genes involved in this process. We suggest that functional analysis of the large group of uncharacterised genes found in our study may contribute to better understanding of the regenerative capacity of cnidarians, as well as provide insight into how wound healing and regeneration has evolved in different lineages.

The extent of an organism’s ability to heal, repair and regenerate damaged tissues varies substantially across eumetazoan lineages. Understanding the molecular mechanisms that underpin these processes has been of long standing interest, particularly as most vertebrate species, upon maturation, adopt a wound healing and regeneration strategy that often results in the loss of original tissue function through scar formation^1^. The mechanism by which wounds heal is distinct from that which controls tissue regeneration; wound healing involves the interplay of factors that attempt to close a wound and prevent infection, often resulting in scar formation^2^, whereas regeneration restores tissue back to its pre-injury state without scar formation^3^. Understanding the processes that determine whether wound healing will progress into regeneration or end as scar formation is not only an important area of clinical research, but is also key to our understanding of the evolutionary loss or gain of regeneration in different eumetazoan lineages^3^.

To date, a number of studies have investigated regeneration in species with high regenerative capacity. Examples include those in the cnidarian *Hydra*^4^, the planarian flatworms^5^, and the urodele salamanders^6^. These species display significant morphological differences, yet certain key molecular mechanisms appear to be present in some or all of these regeneration competent organisms^7^. Brockes and Kumar^7^ summarise many of these mechanisms, which include bioelectrical currents generated by the nervous system, the formation of wound epithelium at the site of injury, the immune system and inflammation, as well as a number of tissue patterning pathways key to embryological development. While studying these similarities contributes greatly to our knowledge of wound healing and regeneration, outlining the differences between these organisms is perhaps of equal importance, in order to determine how this process has evolved in different lineages.

The phylum Cnidaria consists of a diverse range of aquatic species with varied regenerative potential, including anthozoans (corals and sea anemones), scyphozoans (jellyfish), cubozoans (box jellyfish) and hydrozoans. While freshwater *Hydra* may be considered as the model organism for studies of regeneration in Cnidaria, comparative analyses with other cnidarians need to be conducted as more evidence emerges to indicate that the patterns observed in *Hydra* may not be representative of other cnidarian species. For example, the actiniarians *Calliactis parasitica* and *Nematostella vectensis* appear to regenerate despite the absence of stem cells that contribute to regeneration observed in *Hydra*^8^,^9^. Furthermore, the ability to regenerate is not seen in all actiniarians, providing a suitable model to investigate the differences between organisms that have retained this trait and those that have lost it. Given the evolutionary position of Cnidaria as sister group to Bilateria^10^,^11^, it is likely that discoveries made in this group of organisms will affect our perspective on bilaterian regeneration as a whole.

*Calliactis polypus*, in addition to being regeneration competent, is well positioned as a distant relative of the model actiniarian *N. vectensis* and as a closer relative to *Exaiptasia pallida*^12^. *Nematostella vectensis* has been the subject of recent investigations into the transcriptomic response elicited during regeneration^9^,^13^. Additionally, *E. pallida* will serve as an important source of information regarding actiniarian regenerative processes in the future due to its recently published genome^14^. In this study, we assessed the regenerative response of *C. polypus* after catastrophic injury, using a differential gene expression analysis over a course of 96 hours. Our results highlight the role of conserved eumetazoan genes in coordinating wound healing and regeneration in actiniarians, while we also identify the potentially important role of previously uncharacterised lineage-specific genes involved in *C. polypus* regeneration.

## Materials and Methods

### Experimental set-up

Genetically distinct *C. polypus* were obtained from rock pools at Port Cartwright, Sunshine Coast, Australia (refer to Supplementary Data 1 [1. genetic_relatedness]). Animals were housed in glass aquaria with ambient temperatures ranging between 20–22 °C and water conductivity maintained between 33.5 to 35.5 mS/m until experimentation. Four days prior to experimentation, three *C. polypus* individuals were isolated in a separate glass tank and feeding was halted. During the experiment, water quality was maintained at the same conditions as that of the glass aquaria noted previously.

### Animal sectioning

**Figure 1:**
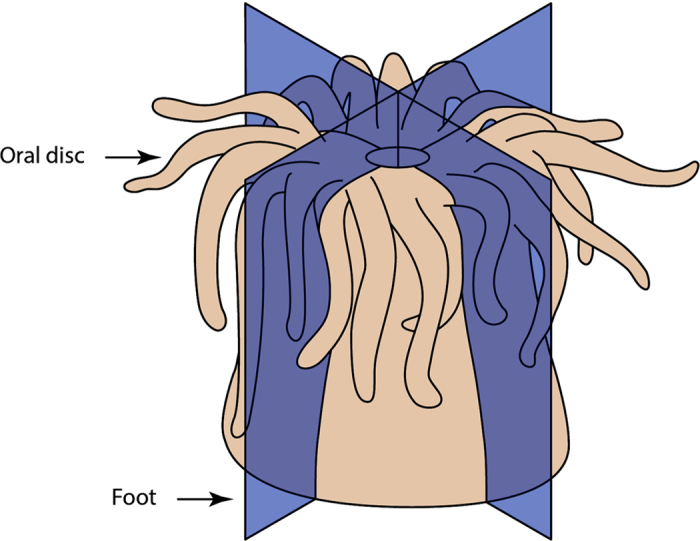
Diagrammatic depiction of the planes through which anemones were cut. Blue coloured longitudinal planes indicate where each *C. polypus* individual (n = 3) was precisely cut from oral disc (top) to foot (bottom), resulting in four equally sized quarters with the same representation of tissues and cell types. Each fragment was sampled to represent a separate time point (0, 3, 20, and 96 hours post sectioning).

To stimulate regeneration, animals were sectioned along their longitudinal axes at right angles, resulting in four equally sized fragments (Fig. 1). It was observed from preliminary experiments that all fragments resulting from this process were able to survive and regenerate into whole organisms. The four fragments originating from each of the three individuals were collected at 0, 3, 20, and 96 hours post sectioning (hps), immediately frozen in liquid nitrogen, and stored at −80 °C for later RNA extraction. These four time points were chosen to reflect physiological and behavioural changes (i.e., at 3 hps the secretion of a mucus layer was evident, and at 96 hps sea anemones began to pulsate their entire bodies) or to reflect stages of regeneration noted in previous studies (i.e., 20 hps marks the early regenerative phase of *N. vectensis*)^9^.

### Transcriptome sequencing and assembly

Total RNA was extracted from each sea anemone fragment after homogenisation in liquid Nitrogen using an RNeasy Midi Kit (Qiagen), with resulting RNA visually inspected on a 1.5% agarose gel stained with GelRed (Biotium). Further quality assessment was performed using an RNA nano chip on a Bioanalyzer 2100 (Agilent) to confirm RNA integrity and concentration. Seventy-five base pair (bp) single-end libraries for each sample were prepared using the Illumina TruSeq Stranded mRNA Library Preparation Kit as per the manufacturer’s instructions, with resulting cDNA quality and concentration being inspected using a high sensitivity DNA chip on a Bioanalyzer 2100 (Agilent). Sequencing was performed using an Illumina NextSeq 500.

Resulting reads were assembled into a single reference transcriptome using the Trinity short-read *de novo* assembler v2.0.6^15^. This process involved the trimming of low quality leading and trailing bases (<Q30) from each read, while also trimming Illumina adapter sequences with the inbuilt Trimmomatic tool^16^. The final reference transcriptome was analysed using BUSCO^17^ to assess the completeness of our transcriptome assembly by identifying the proportion of genes represented in our transcriptome that are expected to be present in almost all metazoans. Additional quality statistics were also obtained, including the number of contigs, N50, and median contig length.

### Differential gene expression

Differential gene expression (DGE) analysis was performed following the Trinity short-read *de novo* assembler’s edgeR protocol^18^. The list of contigs with a statistically significant differential expression (log2 | FC | ≥2, FDR ≤ 0.01, n = 3) was then curated. Curation involved subjecting the differentially expressed contigs to a process of redundancy removal using CD-HIT^19^ to remove duplicated and overlapping sequences with an identity parameter of 95%. After this, the remaining contigs were manually curated using MUSCLE^20^ alignment of contigs assembled as isoforms by Trinity to identify any redundant sequences that were not removed by CD-HIT.

### Conserved open reading frame identification and assembly refinement

**Figure 2:**
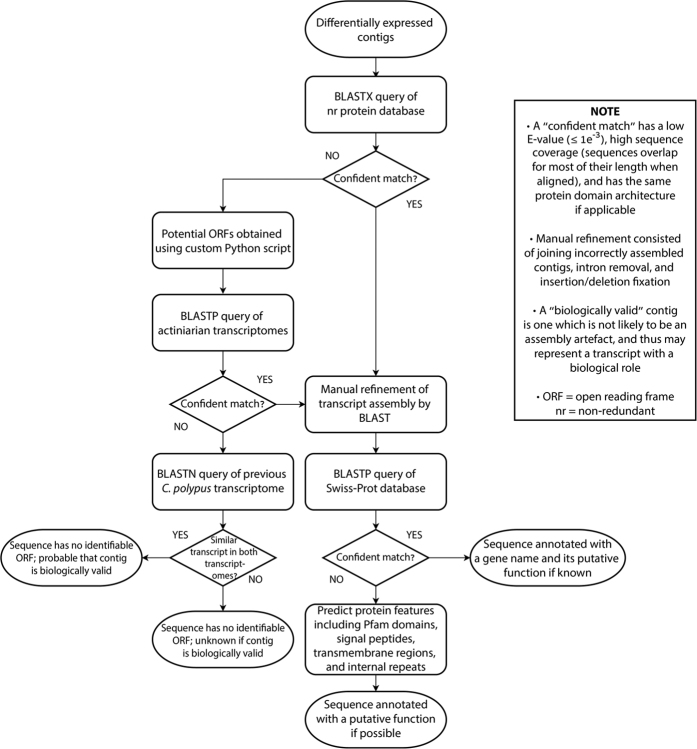
Flowchart depicting the main bioinformatic process employed to identify open reading frames and annotate contigs with functions.

The resulting pool of curated contigs was subjected to a process of open reading frame (ORF) prediction and assembly refinement (Fig. 2). Firstly, protein-coding ORFs were identified by BLASTX query to the non-redundant (nr) protein database^21^ (queried 16/05/16). Contigs lacking ORFs which obtain confidently predicted homologues in the nr protein database (wherein confident refers to a match with a significant E-value [≤1e^−3^], high sequence identity and alignment coverage, and the same protein architecture if applicable) had potential ORFs predicted using a custom Python script (script is available from https://github.com/zkstewart/orf-finder-py). The parameters of this script were set to obtain ORFs with a minimum size of 33 amino acids, with lower stringency for selection of non-canonical ORFs (refer to Supplementary Methods for more detail). These ORFs were utilised as BLASTP queries against a combined database of predicted ORFs identified using the same Python script in the transcriptomes of five actiniarian species. These species included *Nemanthus annamensis, Telmatactis sp*., *Anthopleura buddemeieri, Aulactinia veratra*, and *Actinia tenebrosa* (refer to Supplementary Table S1 for SRA accessions and transcriptome assembly quality scores). Queried *C. polypus* ORFs that obtained confidently predicted homologues (as defined above) in some or all of the other five actiniarians were assumed to represent previously uncharacterised protein-coding regions.

Using the aforementioned database of predicted actiniarian ORFs in conjunction with the nr protein database and a previously assembled *C. polypus* transcriptome (see Supplementary Table S1 for SRA accession and transcriptome assembly quality score), further detailed assembly refinement was performed. This was primarily concerned with manually assembling sequence fragments that had not been correctly assembled by Trinity, the removal of intron sequences in contigs for which we believed evidence suggested no intron should be present, as well as identifying nucleotide insertions or deletions resulting in ORF interruption. The methodology of this is outlined in Supplementary Data 1 (3. assembly_refinement; 4. in_depth_investigation).

### Time point categorisation

Contigs with expression levels that were only found to be statistically different when comparing treatment time points (3 hps, 20 hps, and 96 hps) to each other rather than to control (0 hps) were subjected to further assessment to determine when the contig could be said to differ from physiological baseline expression. To visualise this, scatterplots were created which allowed for inference of physiological baseline expression. This process is detailed further in the Supplementary Methods.

### Functional annotation of contigs

Contigs containing putatively conserved ORFs were functionally annotated (Fig. 2) with reference to proteins in the Swiss-Prot database^22^ when confidently predicted homologues were found. For all other sequences that could not be annotated by Swiss-Prot comparison, translated ORFs had protein features predicted to enable inference of function. This consisted of Pfam^23^ domain prediction (29.0 December 2015 database), as well as features predicted by SMART^24^ which included signal peptides (SignalP 4.1)^25^, transmembrane regions (TMHMM 2.0)^26^, and internal repeats (Prospero)^27^. Contigs that lacked putatively conserved ORFs were compared with a previously assembled *C. polypus* transcriptome (as mentioned above) to find similarly assembled contigs using BLAST, in order to determine the likelihood of these contigs representing genuine biological transcripts. In this, we used stricter parameters than that described above for a “confident” match, requiring the contigs to be similar in overall length, with little to no mismatches and no gap openings outside of the extreme 5′ and 3′ ends.

## Results

### Transcriptome assembly and differential gene expression

A total of 762 million single-end reads originating from 12 samples were generated from this study (SRA accession SRP091618). The *de novo* reference transcriptome consisted of 252, 263 contigs with an N50 of 1,167 bp and median contig length of 385 bp. BUSCO completeness scoring of the *de novo* assembly found 91% of the 843 core metazoan genes (BUSCO summarised benchmark = C: 91% [D: 39%], F: 4.6%, M: 3.6%, n: 843). Individual transcriptomes for each of the 12 samples were also assembled and assessed for completeness using BUSCO, the results of which are presented in Supplementary Table S2.

**Figure 3:**
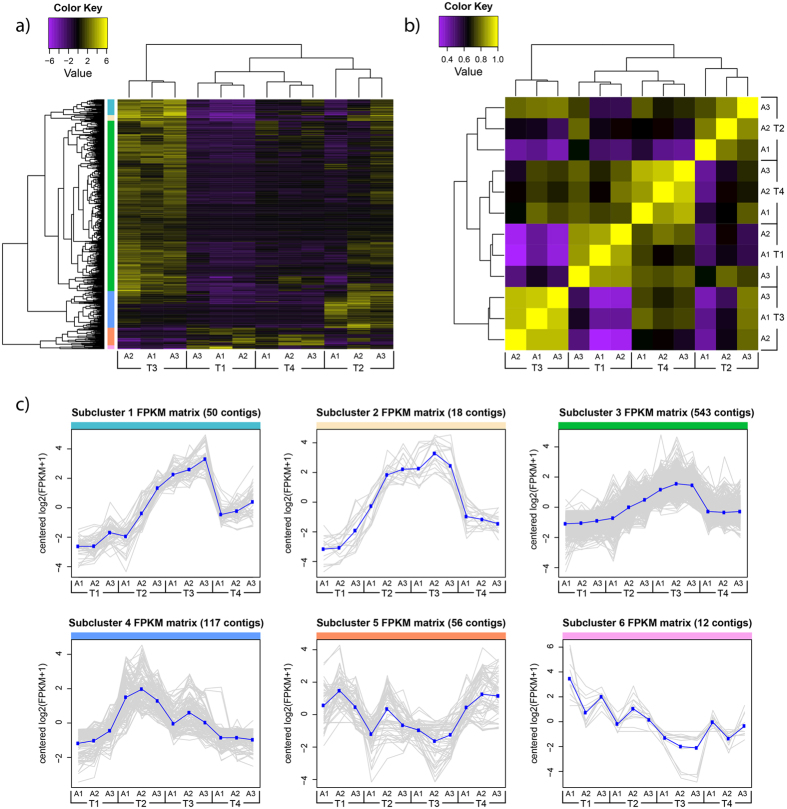
Heat map and sample correlation matrix of differentially expressed genes following injury of *Calliactis polypus*. (**a**) Heat map showing the RNA-Seq expression levels of 796 differentially expressed contigs (log2 |FC| ≥2, FDR ≤ 0.01, n = 3) across the four time points. Expression values are log2-transformed median-centred FPKM. Yellow and purple colour intensity indicate contig upregulation and downregulation, respectively. Dendrogram clustering on the X-axis indicates sample similarity, whereas dendrogram clustering on the Y-axis groups contigs with similar expression profiles over time. Coloured bars on the Y-axis correspond to subclusters shown in (**c**). (**b**) Sample correlation matrix displaying the overall similarity in expression profile for each sample at the four different time points. Yellow colour intensity indicates increasing sample correlation, whereas purple colour intensity indicates decreasing sample correlation. Dendrogram clustering on the X and Y-axis indicates the overall similarity of these samples. (**c**) Contigs with similar RNA-Seq expression patterns were subclustered by cutting the Y-axis dendrogram of (**a**) at 50% of its height, resulting in 6 subclusters. Each contig’s expression, measured in log2-transformed median-centred FPKM, is plotted in grey, with the mean expression profile for each cluster plotted in blue. Coloured bars lining the top of each graph correspond to the sections indicated on (**a**). T1–T4; time 1 – time 4 (0 hrs, 3 hrs, 20 hrs, 96 hours post sectioning), A1–A3; anemone 1 – anemone 3, FPKM; fragments per kilobase of transcript per million mapped reads.

Differential gene expression analysis revealed 796 contigs whose abundance varied over the course of the experiment (Fig. 3a). Sample correlation (Fig. 3b) indicated that time points T1 (0 hps) and T3 (20 hps) were most dissimilar in their contig expression pattern, with T1 and T4 (96 hps) being most similar. Subclustering of differentially expressed contigs with similar RNA-Seq expression patterns was performed by cutting the dendrogram at 50% of its height (Fig. 3a), resulting in 6 subclusters (Fig. 3c).

### Assembly refinement and open reading frame identification

After assembly refinement and redundancy removal, 489 unique differentially expressed contigs remained. Assembly refinement identified 23 incompletely assembled contigs which were manually assembled into eight contiguous transcripts. In addition, we identified four sequences with intron retentions, one misassembled sequence with artefact nucleotide insertion, and one sequence that was full length in an individual transcriptome but not in the combined reference assembly. Through this process, we also removed three putative contaminant contigs. Details of these contigs can be found in Supplementary Data 1 (3. assembly_refinement; 4. in_depth_investigation).

Through the process of ORF identification, 208 contigs contained ORFs with significant identity to sequences in the nr protein database or predicted ORFs from the transcriptomes of the five sea anemone species used for comparison (*N. annamensis, Telmatactis sp*., *A. buddemeieri, A. veratra*, and *A. tenebrosa*), leaving 281 without a predicted ORF. Of the 208 contigs with predicted ORFs, 98 had been compared to predicted ORFs from the five sea anemone transcriptomes to assist in the prediction of the *C. polypus* ORF (refer to Supplementary Data 1 [5. anemone_orfs]). Many of these 98 contigs did not return sequences with significant E-values (≤1e^−3^) when submitted as BLAST queries to the nr protein database. From this we can conclude that these represent ORFs that are novel and likely to be lineage-specific, and suggest that many of these may have been missed during genome annotations of *N. vectensis* and *E. pallida*.

### Time point categorisation

The 489 differentially expressed contigs were categorised as being up or downregulated at 3 hps, 20 hps, and 96 hps when compared to physiological baseline expression. Contigs that did not statistically differ when compared to control expression were assessed to determine if we could categorise them as being up or downregulated when compared to a proposed physiological baseline expression (as detailed in the Supplementary Methods). Four contigs could not be categorised due to their variable expression profile across time points and large variance in expression levels between replicates. These were not considered in downstream analyses, resulting in a final group of 485 contigs.

**Figure 4:**
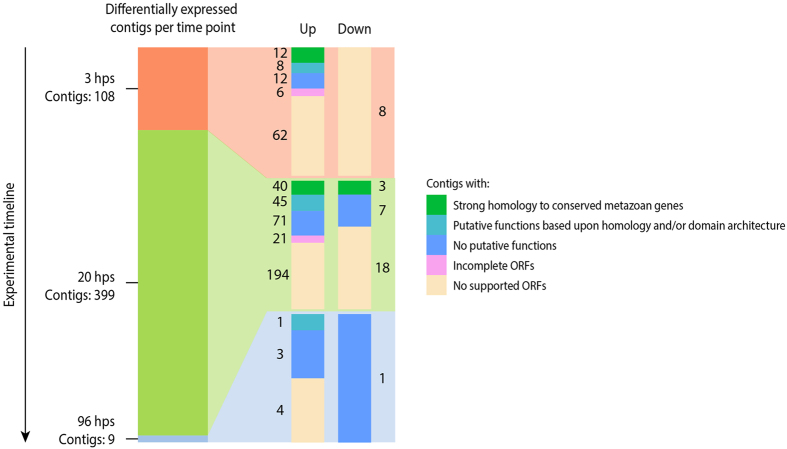
Number and type of differentially expressed contigs at the three treatment time points when compared to baseline expression. The leftmost bar represents the number of differentially expressed contigs at its labelled time point, with the rightmost paired bars representing the number of contigs with relation to their expression status (up or down) and the type of contig as noted in the legend. hps; hours post sectioning, ORF; open reading frame.

Of the 485 differentially expressed contigs, 108, 399, and 9 contigs were categorised as being differentially expressed at 3 hps, 20 hps, and 96 hps, respectively (Fig. 4). Notably, the majority of contigs were upregulated as opposed to downregulated. Moreover, contig differential expression increased greatly from 3 hps to 20 hps, with the number of differentially expressed contigs decreasing markedly by 96 hps. The group of contigs which contained identifiable protein-coding ORFs (208 in total) could be divided into two similarly sized groups with and without annotated functions. The 281 contigs lacking an identifiable protein-coding ORF thus represent a large proportion of the differentially expressed contigs, for which no functional roles can be inferred.

### Functional annotation of contigs

#### Genes of interest

Functional annotation of the differentially expressed contigs identified a number of genes with potentially important roles in wound healing and regeneration within *C. polypus*. This included many genes with significant identity to known transcription factors involved in tissue patterning, such as *Sox E1, Bicaudal*-*C homologue 1 (BICC1*), *Doublesex* and *mab*-*3*-*related transcription factors A* and *F (DMRT A*/*F*), *Pax D2*, and *Nuclear factor 1 X*-*type (NF1X*), as well as uncharacterised proteins with domain architecture indicative of transcription factor activity (PF00170: bZIP domain, PF01342: SAND domain, zinc finger domains with high identity to known zinc finger transcription factor domains). In addition to these, we also identified genes involved in fibroblast growth factor (FGF) signalling, namely a FGF protein and the FGF feedback inhibitor *Sprouty*.

Stress response and wound healing genes were represented in our study, including *Biliverdin reductase A, DnaJ*-*like 1*, and *Heat shock factor 1*, and *Hspa12B*.

Genes involved in cellular energy dynamics included *Solute carrier family 25 member 36*-*like, Graves’ disease carrier protein, Phosphoenolpyruvate carboxykinase* [*GTP*], *LYR motif*-*containing protein 5, Thiopurine S*-*methyltransferase, Amidophosphoribosyltransferase*, and *PAICS*.

Genes proposed to be involved in immunity were differentially expressed, including *Alpha*-*2*-*macroglobulin (A2M*), two putative interleukin-1 receptor-like genes, and a number of genes with putative antimicrobial roles such as *Autophagy*-*related protein LC3C, Fibrocystin*-*like* gene (*PKHDL1*), as well as those containing domains commonly associated with immune responses (PF00530: Scavenger receptor cysteine-rich, PF04505: Interferon-induced transmembrane protein).

Genes related to cell signalling also represented a large proportion of functionally annotated contigs, including two adhesion G protein-coupled receptors, three Ras-related small GTPases, the calcium signalling molecule *Calmodulin*, and eight uncharacterised proteins with receptor-related protein domain (PF00001: 7 transmembrane receptor [rhodopsin family]), five of which shared moderate identity to known neurotransmitter receptors annotated on UniProtKB/Swiss-Prot (e.g., dopamine receptors, alpha and beta adrenergic receptors, galanin receptors, among others).

Finally, genes involved in extracellular matrix-related functions were identified, which included a putative cnidarian homologue of the type 1 collagen synthesising *La*-*related protein 6 (LARP6*), in addition to three tissue inhibitors of metalloproteinase (TIMP) and four A disintegrin and metalloproteinase with thrombospondin motif (ADAMTS) proteins.

**Figure 5:**
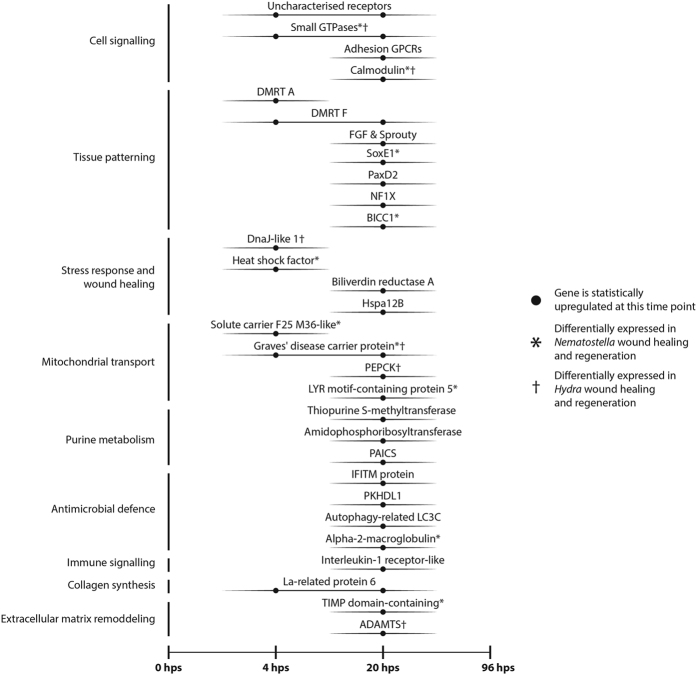
Key candidate genes identified through our differential gene expression study likely to be important for wound healing and regeneration. Note that no contigs with putative functions were found to be differentially expressed at 96 hours post sectioning (hps). Genes noted with an asterisk (*) or dagger (†) were also differentially expressed in a study of *N. vectensis*^9^ or *Hydra*^28^, respectively. Abbreviations - Adhesion GPCR; Adhesion G protein-coupled receptor, DMRT A/F; Doublesex and mab-3 related transcription factor A/F, FGF; Fibroblast growth factor, Sox E1; Sry-related HMG box E1, PaxD2; Paired box D2, NF1X; Nuclear factor 1X-type, BICC1; Bicaudal-C homologue 1, Hspa12B; Heat shock protein family A member 12B, Solute carrier F25 M36-like; Solute carrier family 25 member 36-like, PEPCK; Phosphoenolpyruvate carboxykinase, PAICS; Phosphoribosylaminoimidazole carboxylase/phosphoribosylaminoimidazole succinocarboxamide synthetase, IFITM protein; Interferon induced transmembrane protein, PKDHL1; Polycystic kidney and hepatic disease-like 1 (or ‘Fibrocystin-like’), Autophagy-related LC3C; Autophagy-related protein light chain 3 C, TIMP domain-containing; Tissue inhibitors of metalloproteinase domain-containing, ADAMTS; A disintegrin and metalloproteinase with thrombospondin motifs.

The differential expression patterns of these genes are summarised in Fig. 5, and the full annotation report is available as Supplementary Data 1 (2. annotation_report).

#### Uncharacterised genes

The contig with the largest fold change when compared to control, as well as the highest overall log2-transformed median-centred expression level (three replicate average ≈ 3.85), was an upregulated, uncharacterised sterile alpha motif domain-containing protein (PF07647: SAM domain). This was followed by the immune-related *A2M*, which had the third largest fold change when compared to control and second highest overall log2-transformed median-centred expression level (three replicate average ≈ 3.67). The remaining contigs in the top 10 according to either of these two metrics (i.e., fold change compared to control, and log2-transformed median-centred expression level at a specific time point) were all uncharacterised sequences without putative functions. It is worth noting, however, that within the top 10 of these two metrics were a group of uncharacterised genes with a predicted protein domain which is typically associated with prokaryotic enterotoxins (PF05791: *Bacillus* haemolytic enterotoxin). This domain, to our knowledge, has not been previously annotated in any eukaryotic proteins on the nr protein database (as of 16/05/16) with the exception of two proteins in *E. pallida* (KXJ22104, KXJ22064) and one in *Acropora digitifera* (XP_015750752), both of which are cnidarians. Based upon preliminary bioinformatic evidence, these are not likely to represent contaminants in our data (refer to Supplementary Data 1 [4. in_depth_investigation]).

For those contigs where we could not identify putatively conserved ORFs (281), we found 71 similarly assembled contigs in a previously assembled *C. polypus* transcriptome, representing approximately 25% of this group of contigs. Refer to Supplementary Data 1 (7. no_orf_check) for a list of these contigs.

## Discussion

In our study we have compiled a rich dataset and identified a large number of candidate genes involved in wound healing and regeneration in the sea anemone *C. polypus*. Manual annotation of contigs reveals that conserved genes from various metabolic pathways are differentially expressed in *C. polypus* after wounding. These include genes related to tissue patterning and embryogenesis, stress response, energy dynamics, the immune system, cell signalling, and extracellular matrix (ECM) remodelling (Fig. 5). Most of these genes were upregulated after wounding, with very few downregulated genes identified to which we could attribute functional roles (Fig. 4). We also observe a large number of uncharacterised, putatively lineage-specific genes, that could not be functionally annotated (Fig. 4). We propose that wound healing and regeneration in *C. polypus* is enabled by a combination of conserved genes as well as genes that are specific to Cnidaria and, possibly, to Actiniaria.

### Time point selection

Overall, the time points we have selected for sampling have provided us with baseline data related to the genes involved in *C. polypus* regeneration. Sampling at 3 hps was chosen due to secretion of a mucus layer over the wound. While we don’t find evidence of changes in expression of transcripts typically considered to be involved in mucus secretion at 3 hps, we do identify a mucin-like gene that is downregulated at 20 hps. This gene may be involved in the production of mucus within the first few hours of *C. polypus* regeneration. We find that 20 hps represents a time of heightened transcriptomic activity, characterised by genes involved in a variety of different cellular pathways. At 96 hps, we did not find any transcripts that could explain the observed pulsating behaviour. We do, however, note that baseline expression levels are largely restored by this time point (Fig. 4). This is significant, as the morphology of the organism indicates that wound healing and regeneration are still occurring after 96 hps (Supplementary Figs S1 and S2). Based on this observation, we hypothesise that a transition from transcriptomic activity to proteomic activity may occur between 20 hps and 96 hps. If this is the case, future studies of cnidarian regeneration should use proteomic techniques in combination with RNA-Seq analysis.

### Conserved cnidarian regenerative properties

Comparison of our *C. polypus* study to two previous studies^9^,^28^ of cnidarian regeneration using transcriptomic approaches revealed key mechanisms that appear to be conserved throughout Actiniaria and Cnidaria (see Supplementary Data 1 [6. comparison_regen_studies]). Unsurprisingly, the differentially expressed genes we identify in *C. polypus* most closely correspond to the gene cohort reported by DuBuc *et al*.^9^ in *N. vectensis* regeneration, with a smaller overlap when compared to Petersen *et al*.’s^28^ study of *Hydra*. Genes found to be differentially expressed in all three species include a Ras-related small GTPase as well as *Calmodulin*, highlighting the role of intracellular signal transduction during regeneration. We also note that *Calmodulin* is upregulated in rat liver regeneration models^29^, which indicates a potentially conserved role of *Calmodulin* in regenerative processes throughout Eumetazoa. Finally, we find a putative homologue of the *Graves’ disease carrier protein* which, based upon evidence from human and yeast models^30^, might play an important role in accumulating coenzyme A in the mitochondria of these three animals. Genes differentially expressed in *C. polypus* and either *N. vectensis* or *Hydra* also include those related to tissue patterning, stress responses, mitochondrial transport, antimicrobial defence, and ECM remodelling (Fig. 5).

The genes noted in Fig. 5 are not the only genes we find in common between *C. polypus* and *N. vectensis* or *Hydra*. Other contigs without ascribed functions include a putative *Protein phosphatase inhibitor*-*2* and a cohort of uncharacterised genes. One such uncharacterised gene contains a PF00170: bZIP domain, suggestive of transcription factor activity.

### Overview of genes involved in *Calliactis polypus* regeneration

#### Early response

Contigs identified in subclusters 2 and 4 (Fig. 3c) appear to represent a group of genes whose expression increases immediately following severe wounding. A high proportion of these genes are putative transcription factors or are involved in cellular stress responses, such as heat shock proteins. The early upregulation of stress response genes is expected given the severity of wounding during the experiment. With respect to transcription factors, we hypothesise that many of these may coordinate the regulation of genes differentially expressed at 20 hps, which are represented in subclusters 1, 3, and 6 (Fig. 3c).

Genes involved in the early response of *C. polypus* regeneration also appear to be highly upregulated. Subcluster 2 contains many of the uncharacterised genes within the top 10 of the two expression metrics mentioned in the Results, which includes the SAM domain-containing protein and two of the three *Bacillus* haemolytic enterotoxin (PF05791) domain-containing proteins. While it is difficult to assign any specific function to these genes, we postulate that genes that are highly upregulated immediately and remain elevated later into the regeneration response may be of particular importance for *C. polypus* and, more generally, cnidarian regeneration. Subsequently, we believe that these genes in particular deserve further attention, as they may provide unanticipated insights into cnidarian regeneration.

#### Later response

The largest number of differentially expressed contigs was observed at 20 hps (subclusters 1, 3, and 6). While most of the cellular pathways identified in the transcriptomic response of *C. polypus* at this time point are also reported in other cnidarian models (Fig. 5), there are certain cellular pathways and genes of interest which our results suggest may hold additional significance. The genes and pathways of interest include tissue patterning, mitochondrial transport and energy dynamics, innate immune system and ECM remodelling genes.

A number of upregulated tissue patterning genes were identified 20 hps, including *DMRT A* and *F*, for which evidence suggests the paralog *DMRT B* is involved in neurogenesis^31^. Additionally, we find the gene *Pax*-*D2* to be upregulated, which in *N. vectensis* is involved in neurogenesis^32^. Furthermore, we identify a FGF protein and the FGF feedback inhibitor *Sprouty* as upregulated, both of which are expressed in *N. vectensis* embryos and are thought to be involved in endoderm formation and neurogenesis^33^. Taken together, these genes indicate that neurogenesis is occurring during *C. polypus* regeneration. In consideration of these and other genes involved in coordinating tissue patterning processes that are upregulated at 20 hps (i.e., *Sox E1, BICC1, NF1X*), we also propose that *C. polypus* may be reverting back to an embryogenesis-like state, wherein whole-body tissue patterning processes are recapitulated to enable regeneration.

Genes involved in mitochondrial transport and energy dynamics appear to play a role in *C. polypus* regeneration (Fig. 5). While the relationship between mitochondrial energy production and regeneration has not been well established in the literature, the results of our study and another recently published study on neuronal axon regeneration^34^ suggest that understanding this relationship may be important for understanding regeneration as a whole. Further genes that indicate a change in *C. polypus* energy dynamics include those involved in purine metabolism. We observe the upregulation of two genes involved in *de novo* purine synthesis, including *Amidophosphoribosyltransferase* and *PAICS*. It is noteworthy that we do not find genes involved in salvage synthesis of purines, the method of purine synthesis typically employed in cells, to be differentially expressed. Increases in *de novo* synthesis of purines have been observed within *Xenopus* tadpole tail regeneration^35^ as well as in rat liver regeneration^36^. Given its implication in changes from oxidative phosphorylation to glycolysis to meet energetic demands^37^, transcriptomic data of *C. polypus* regeneration might indicate a shift towards glycolysis. This has been proposed in the *Xenopus* regeneration model^35^ which, in conjunction with our findings, suggests a conserved role for *de novo* purine synthesis and glycolysis in eumetazoan regeneration.

Genes with a role in the innate immune system appear to have a slower activation in *C. polypus* in response to injury. Two interleukin-1 receptor-like proteins, involved in coordinating the activation of the innate immune system^38^, are upregulated at 20 hps. We also see *A2M* upregulated, a gene that was differentially expressed in DuBuc *et al*.’s^9^ study on *N. vectensis* but not in Petersen *et al*.’s^28^ study of *Hydra*. Due to its high level of differential expression, *A2M* is likely to have an important role in modulating the immune response during regeneration in actiniarians.

In our study, ECM remodelling genes also constitute a major cellular pathway observed in the later transcriptomic response of *C. polypus*. Representatives of this pathway include TIMPs as well as ADAMTS genes, both of which represent gene families with various functions in ECM remodelling^39^,^40^. In addition, we report up-regulation of the cnidarian homologue of *LARP6*. This gene has been shown to result in an increase in type I collagen protein synthesis without affecting type I collagen mRNA abundance^41^. The potential increase in synthesis of type I collagen indicated in the transcriptome of *C. polypus* is in line with a previous histological study of the related *C. parasitica*, where increased amounts of collagen filaments were observed during wound healing^8^. Thus, while we do not detect any differentially expressed collagen transcripts in our study, our results corroborate this observation.

### The role of uncharacterised genes in regeneration and wound healing

Our study has revealed a high proportion of differentially expressed contigs with uncharacterised ORFs (Fig. 4). These uncharacterised ORFs likely represent important lineage specific genes involved in regeneration in cnidarians. An increased scientific effort to functionally characterise the many genes found only in cnidarians^42^,^43^ will be necessary for future efforts to understand regeneration in this phylum.

It is also important to note the large number of contigs (281) for which we could not find conserved ORFs. In an attempt to discern whether these contigs were likely to represent either sequencing artefacts or biologically valid transcripts, we found that 71 (~25%) of these had good matches to a previously assembled *C. polypus* transcriptome. Our hypothesis is that many of these contigs are likely to encode small ORFs. The study of small ORFs represents a relatively new field that has only recently been explored in animals and plants utilising ribosomal profiling techniques^44^,^45^. Future studies on cnidarian regeneration utilising ribosomal profiling may provide clues into the role of small ORF-coding transcripts in regeneration and wound healing processes.

### Limitations

It should be mentioned that a limitation of our study was that a sample collected 3 hps contained some degree of ribosomal contamination, which resulted in a poorer representation of genes and may have impacted the statistical ability to detect differentially expressed genes at this time point (refer to Supplementary Table S2). Importantly, a limitation in non-model species is that results often rely on inferring homology to genes studied in models such as human, mouse, or zebrafish. Given the large evolutionary distance between cnidarians and these models (cnidarians originated ~686–819 million years ago)^46^ it would not be surprising if some of the functions we have tentatively attributed to certain genes show some divergence.

## Conclusion

In conclusion, our results indicate that wound healing and regeneration in *C. polypus* involves a large-scale response at the transcriptomic level. Many of the genes identified in our study can be broadly classified into key gene groups, such as those involved in tissue patterning, stress responses, cellular energy dynamics, the immune system, and ECM remodelling. For the sake of brevity, we have only discussed certain genes which provide an encompassing view of the overall processes likely to be involved in *C. polypus* regeneration. From this, we have been able to generate a number of important hypotheses for future regeneration research to investigate in both cnidarians and other animal groups, the results of which should deepen our understanding of how regeneration occurs in certain lineages.

## Additional Information

**Accession codes:** Raw sequencing reads generated from this study are deposited at the NCBI sequence read archive (SRA) under accession SRP091618.

**How to cite this article**: Stewart, Z. K. *et al*. Transcriptomic investigation of wound healing and regeneration in the cnidarian *Calliactis polypus.* *Sci. Rep.* **7**, 41458; doi: 10.1038/srep41458 (2017).

**Publisher's note:** Springer Nature remains neutral with regard to jurisdictional claims in published maps and institutional affiliations.

## Supplementary Material

Supplementary Information

Supplementary Dataset 1
