# Supplementary material for: Transcriptomic investigation of wound healing and regeneration in the cnidarian Calliactis polypus
Source: Sci Rep. 2017 Feb 2;7:41458. doi: 10.1038/srep41458 (PMC5288695; doi:10.1038/srep41458)
Supplement: Supplementary Information [file srep41458-s1.pdf]

Transcriptomic investigation of wound healing and regeneration in the cnidarian *Calliactis polypus*

Zachary K. Stewart[1*], Ana Pavasovic[2,3], Daniella H. Hock[4], and Peter J. Prentis[1,5]

[1]School of Earth, Environmental and Biological Sciences, Queensland University of Technology

[2]School of Biomedical Sciences, Queensland University of Technology

[3]Institute of Health and Biomedical Innovation, Queensland University of Technology

[4]Biological Sciences Centre, Federal University of Santa Catarina

[5]Institute for Future Environments, Queensland University of Technology

*** Corresponding author**

Email: zkstewart1@gmail.com

Address: Queensland University of Technology, GPO Box 2434, Brisbane, QLD 4001

Supplementary Methods

Python script description

‘biopython_orf_find.py’

ORF = open reading frame

Refer to https://github.com/zkstewart/orf-finder-py to download the script.

Dependencies

This script was designed to work with Python versions 3.4 and 3.5, and utilises the in-built ‘**re**’ and ‘**os**’ packages, as well as the external ‘**Biopython**’ package (https://github.com/biopython/biopython.github.io/). This script was built and tested on the Windows 10 OS, though there should be no reason why it cannot run on any OS that Python is capable of running on.

Description of script logic

The custom Python script used in this study is the creation of Zachary K. Stewart. This script was designed to be used by those unfamiliar with command line operations. Thus, the starting section of the script has text prompts which specify to the user what commands are required at each point, with checks in place to ensure the user inputs the correct values. The order of this is to specify the name of the fasta file which contains the nucleotide sequences from which ORFs will be extracted, followed by the output file name which will contain the extracted ORFs, the minimum ORF length you wish to consider, the number of ORFs you wish to obtain which meet this length requirement, and two stringency values which will determine the weighting with which we will consider ORFs with non-canonical (i.e., TTG, GTG, CTG) or no-codon (i.e., fragmented sequence) starts as opposed to traditional (i.e., ATG) start sites. Before delving into the specifics of how these stringencies work, it should first be mentioned that this script works on the basis of identifying regions in-between stop codons. Thus, to this script, an ORF is a region uninterrupted by stop codons. Returning to the stringency values, these values have defaults which I recommend the script runs with, but if shorter peptides (such as those of 10-50AA length) which often have alternative starts are sought, then lowering the stringency of these default values manually is a valid option. No-codon starts are by default weighted against the most heavily, as the assumption with this is that the transcript being analysed is fragmentary and lacks its actual start site. This assumption should only be considered in the absence of any identifiable, putative ORFs with

start codons within the sequence, hence the heavy weighting. The two stringency values together help to define the 'best' ORF identified in a sequence according to its overall length. As such, while an alternative start may render a longer ORF than an ATG start, the stringency value will weight this so that a non-canonical start will only be accepted if it increases ORF length by a large (or user specified) margin. As such, this script works solely on the basis of ORF length, attempting to provide the longest ORF with the most sensible start codon.

File in- and output

This script will read in fasta-formatted '**.fa'** or '**.fasta'** files containing nucleotide sequences. The output will be a fasta-formatted '**.fasta**' file containing protein translations of ORFs identified. The original sequence identifiers will be modified in this output to contain the ORF number as determined from this script. For example, if an original nucleotide sequence is titled '**>contig1**', depending on the number of ORFs identified in this sequence, the output file will have entries titled '**>contig1_ORF1**' and '**>contig1_ORF2**', etc.

Additional notes

As the writer of this script is self-taught, this script may not necessarily conform with writing standards such as PEP8, which may reduce its readability. The script should be relatively light on CPU and RAM usage, and thus should be suitable for use on all types of computers. Unless the CPU of the computer running this script is very weak, this script should be capable of processing files with hundreds of thousands of sequences in time spans of less than 10 minutes (approximately), though depending on user settings (such as how small of a minimum ORF length you specify or how many ORFs you extract from each sequence) this time can vary to some degree. As this script regularly updates the user on the progress of the script, it can be roughly gauged how long the script should take to complete.

More complex ORF finders may often consider things such as GC content and the presence of Kozak consensus sequences among other features. Due to the ability to determine the strictness with which we consider alternative starts, the script is designed to be suitable for finding novel ORFs wherein assumptions of GC content and other sequence features may not hold. Additionally, as this script is capable of pulling many ORFs out of a sequence, it is also intended for performing analyses such as the one in this study, wherein multiple transcriptomes had potential ORFs extracted and compared via BLAST to identify conserved regions. Subsequently, as mentioned, this script is designed primarily with novel ORF identification in mind. If you intend to use this for yourself, you may want to consider what

your goals are, as this script is not necessarily designed to find the most biologically valid start codon of conserved genes, which typically demonstrate certain sequence features.

Script usage in this study

In this study the transcriptomes of *Calliactis polypus* (originating from this study), as well as *Nemanthus annamensis*, *Telmatactis sp.*, *Anthopleura buddemeieri*, *Aulactinia veratra*, and *Actinia tenebrosa* had ORFs extracted using this script. Following the order of inputs detailed in ‘Description of script logic’, these species had their transcriptome fasta files read in, an output file name associated with this, a minimum ORF length of **33AA** specified, **5** ORFs extracted which meet this minimum length, and a modified stringency value of **34AA** length increase to accept an alternative start, and **69AA** length increase to accept a no-codon start (as opposed to the default **49AA** and **99AA**, respectively). It was decided to use less strict stringency values as the intention was to find potentially novel ORFs, which may not necessarily utilise a traditional start codon. Additionally, the fact that we did not find confident matches to the nr database might indicate fragmentation, hence the lower no-codon stringency. This was balanced against the assumption that even a novel ORF, if conserved between any of the sea anemone species assessed here, is still likely to have a traditional start as it will have been conserved over potentially long evolutionary times. The resulting output files of ORFs were then used in local BLAST analyses, the results of which are presented in Supplementary Data 1 (5. anemone_orfs).

Time point categorisation

The 489 differentially expressed transcripts were categorised as being up or downregulated at 3 hours post sectioning (hps), 20 hps, and 96 hps when compared to baseline. For most transcripts, baseline was taken to be expression in control (0 hps); as some of these transcripts were only found to differ statistically when compared to time points other than control, further assessment of these was performed to categorise these transcripts. This treatment was required for 83 transcripts. For these cases, we wished to see if it would be sensible to consider the expression levels of one of the treatment time points (i.e., 3 hps, 20 hps, or 96 hps) to represent baseline expression. The method by which we did this was to create scatterplots of each transcript's log2-transformed median-centred expression values to observe patterns in the data which would allow us to infer what the baseline level of expression was. The logic that went into this process is demonstrated by way of two examples below, with the complete list of scatterplots also attached to this document.

How to read figure: Each dot (three dots per time point) represents the log2-transformed median-centred expression value for one of the biological replicates. The black line joining each dot within a single time point highlights the range of values for each time point's replicates. The red line indicates the mean value of the biological replicate's expression values across the four time points. With relation to this analysis, gap regions where no time point's ranges (black lines) overlap are significant for indicating when a time point is differentially expressed.

Example 1 (positive attribution of baseline status) - TR39823|c0_g2_i1:

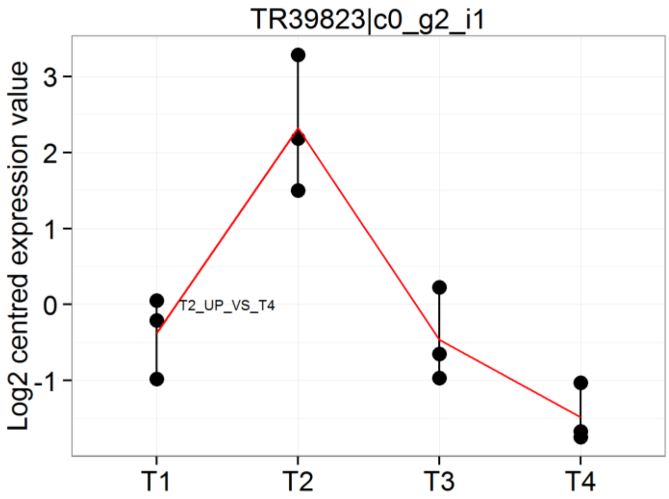

As indicated on this scatterplot, the only two time points that differ statistically are T2 (3 hps) and T4 (96 hps). This presents a problem, as physiological baseline expression of a transcript would be expected to be represented by T1 (0 hps, control). However, the scatterplot does demonstrate that there is an unambiguous and sharp rise in expression at T2 when compared to all three other time points (i.e., the range of values for T2 do not overlap with the other time points). Thus, we believe that we can suggest that this transcript's expression is **upregulated** at T2, with the relative expression indicated at T4 being considered to be **baseline** as it overlaps the values at time points T1 and T3.

Example 2 (negative attribution of baseline status) - TR80314|c0_g1_i1:

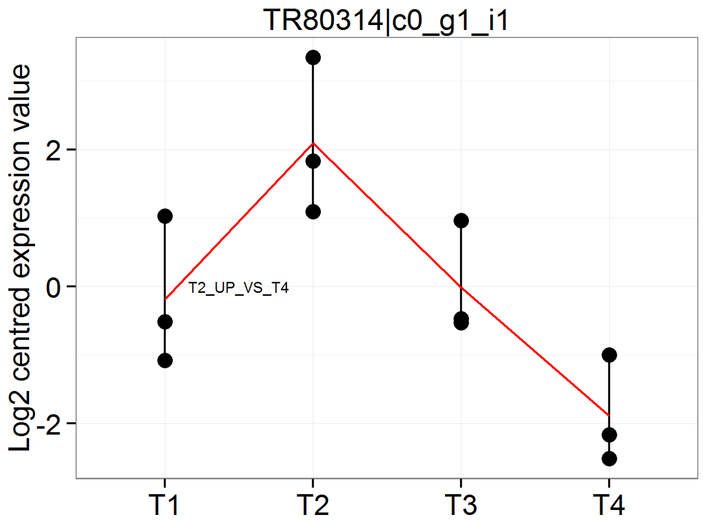

As indicated on this scatterplot, the only two time points that differ statistically are T2 (3 hps) and T4 (96 hps). Similar to the above discussed example, we can see a rise in expression at T2. However, this rise is **not unambiguous**, as the range of data points overlap among time points T1, T2, and T3. T4 has a largely reduced average expression (red line) when compared to the other three time points, but it still overlaps with T1 (which itself overlaps with T2 and T3). Thus, because there is no time point which is clearly differentiated by not overlapping with any other time points, we opted to not declare this transcript as being differentially expressed specifically at one time point as it is unsure if the transcript is being upregulated at T2, downregulated at T4, or both.

The same process of reason depicted in the above two examples was used for all of the 83 transcripts that did not statistically differ when compared to control. The conclusions made for each of these transcripts are noted in the annotation report supplementary file (Supplementary Data 1 [2.annotation_report]). Scatterplots for the 83 transcripts are presented below.

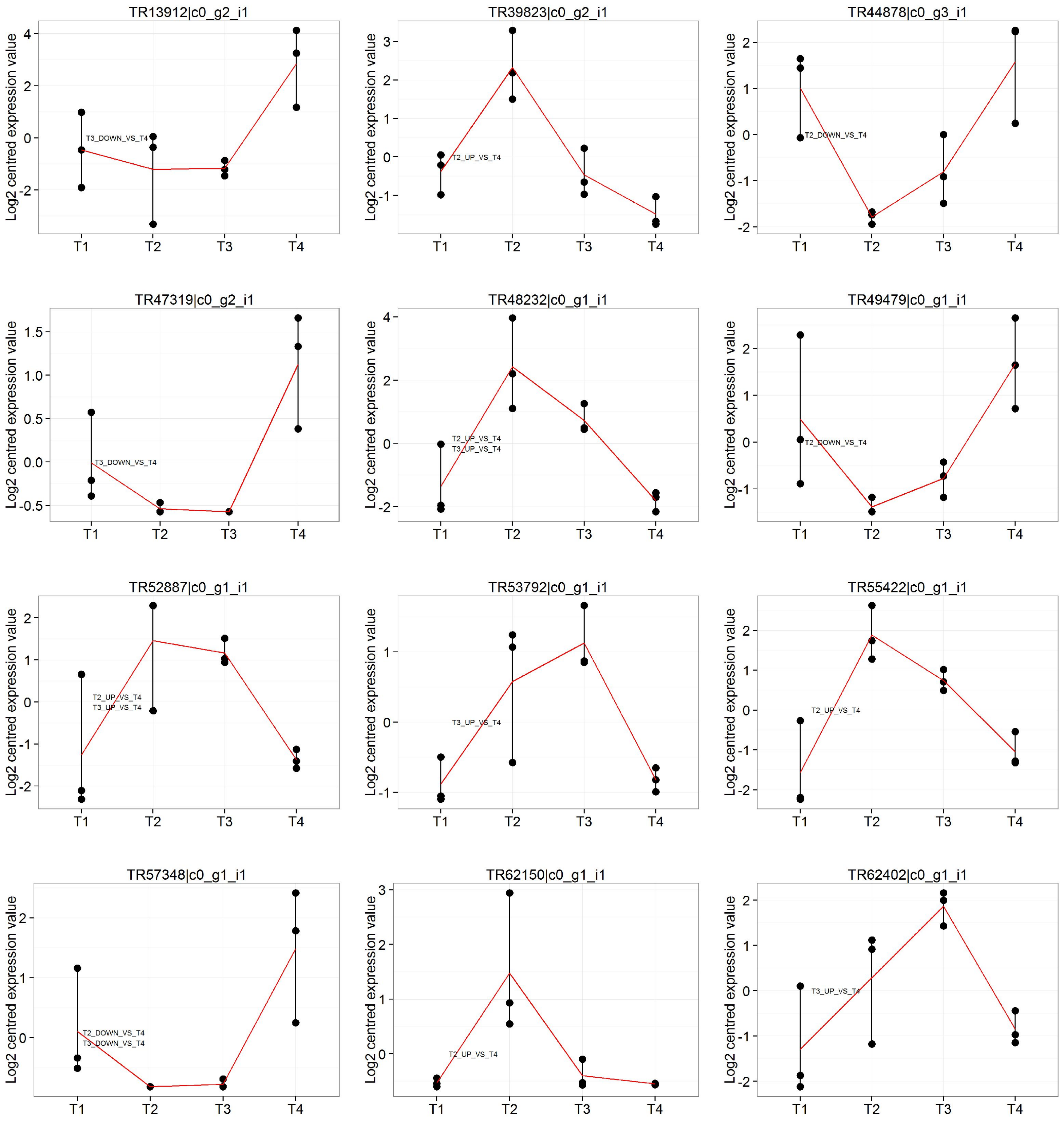
TR13912|c0_g2_i1
T3_DOWN_VS_T4
TR39823|c0_g2_i1
T2_UP_VS_T4
TR44878|c0_g3_i1
T2_DOWN_VS_T4
TR47319|c0_g2_i1
T3_DOWN_VS_T4
TR48232|c0_g1_i1
T2_UP_VS_T4
T3_UP_VS_T4
TR49479|c0_g1_i1
T2_DOWN_VS_T4
TR52887|c0_g1_i1
T2_UP_VS_T4
T3_UP_VS_T4
TR53792|c0_g1_i1
T3_UP_VS_T4
TR55422|c0_g1_i1
T2_UP_VS_T4
TR57348|c0_g1_i1
T2_DOWN_VS_T4
T3_DOWN_VS_T4
TR62150|c0_g1_i1
T2_UP_VS_T4
TR62402|c0_g1_i1
T3_UP_VS_T4
Log2 centred expression value
T1
T2
T3
T4

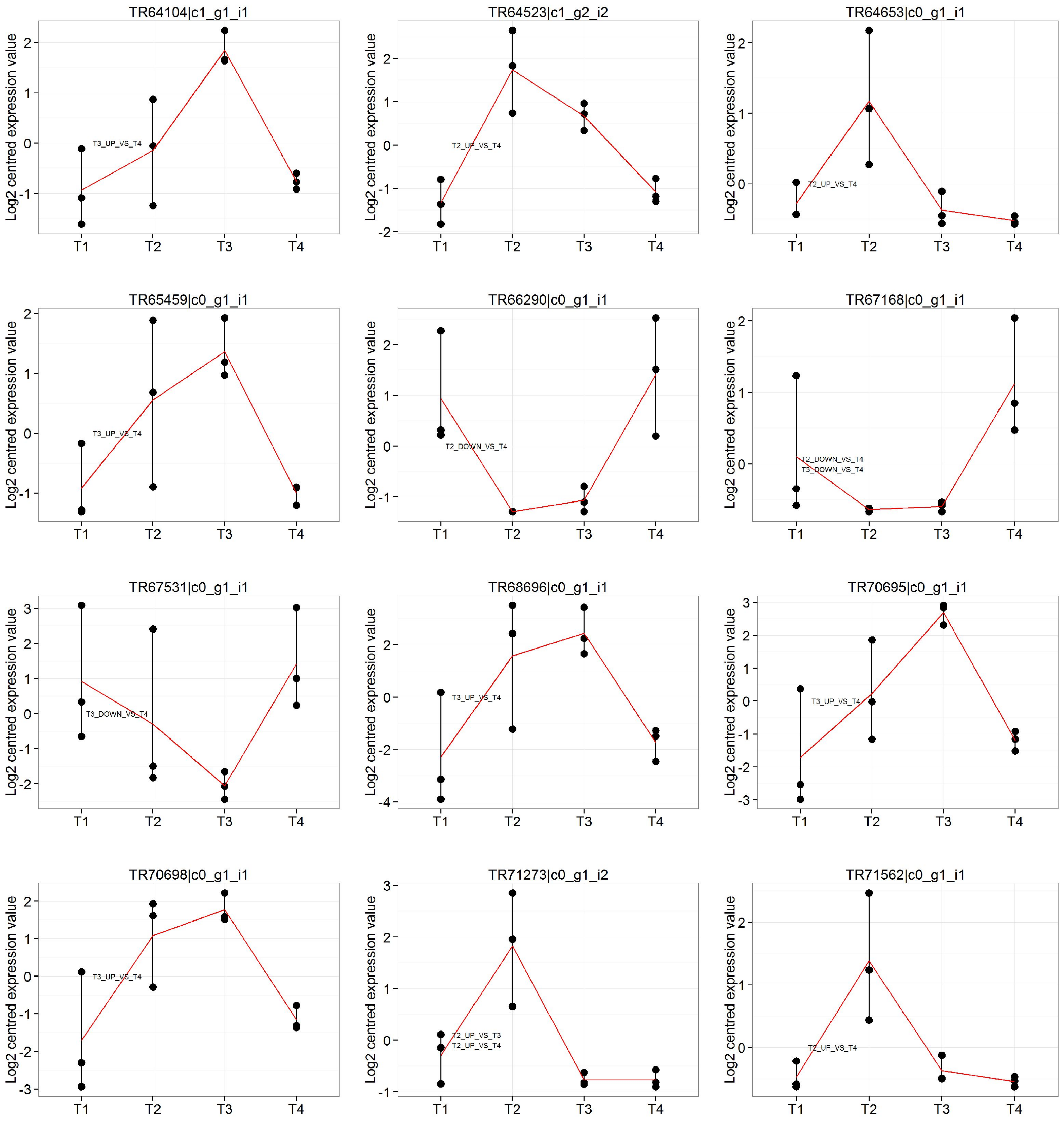
TR64104|c1_g1_i1
T3_UP_VS_T4
TR64523|c1_g2_i2
T2_UP_VS_T4
TR64653|c0_g1_i1
T2_UP_VS_T4
TR65459|c0_g1_i1
T3_UP_VS_T4
TR66290|c0_g1_i1
T2_DOWN_VS_T4
TR67168|c0_g1_i1
T2_DOWN_VS_T4
T3_DOWN_VS_T4
TR67531|c0_g1_i1
T3_DOWN_VS_T4
TR68696|c0_g1_i1
T3_UP_VS_T4
TR70695|c0_g1_i1
T3_UP_VS_T4
TR70698|c0_g1_i1
T3_UP_VS_T4
TR71273|c0_g1_i2
T2_UP_VS_T3
T2_UP_VS_T4
TR71562|c0_g1_i1
T2_UP_VS_T4
Log2 centred expression value
T1
T2
T3
T4

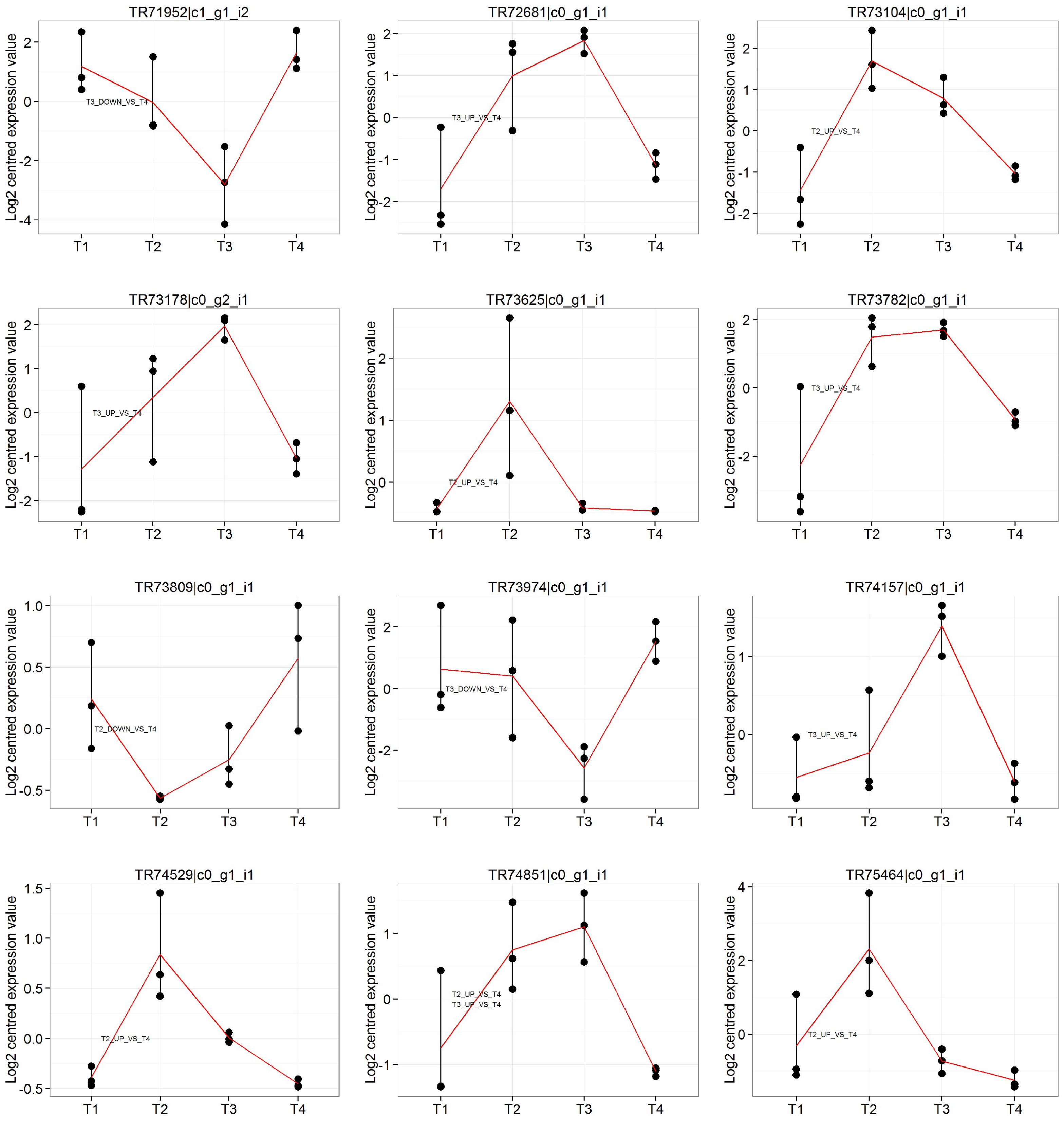
TR71952|c1_g1_i2
T3_DOWN_VS_T4
TR72681|c0_g1_i1
T3_UP_VS_T4
TR73104|c0_g1_i1
T2_UP_VS_T4
TR73178|c0_g2_i1
T3_UP_VS_T4
TR73625|c0_g1_i1
T2_UP_VS_T4
TR73782|c0_g1_i1
T3_UP_VS_T4
TR73809|c0_g1_i1
T2_DOWN_VS_T4
TR73974|c0_g1_i1
T3_DOWN_VS_T4
TR74157|c0_g1_i1
T3_UP_VS_T4
TR74529|c0_g1_i1
T2_UP_VS_T4
TR74851|c0_g1_i1
T2_UP_VS_T4
T3_UP_VS_T4
TR75464|c0_g1_i1
T2_UP_VS_T4
Log2 centred expression value
T1
T2
T3
T4

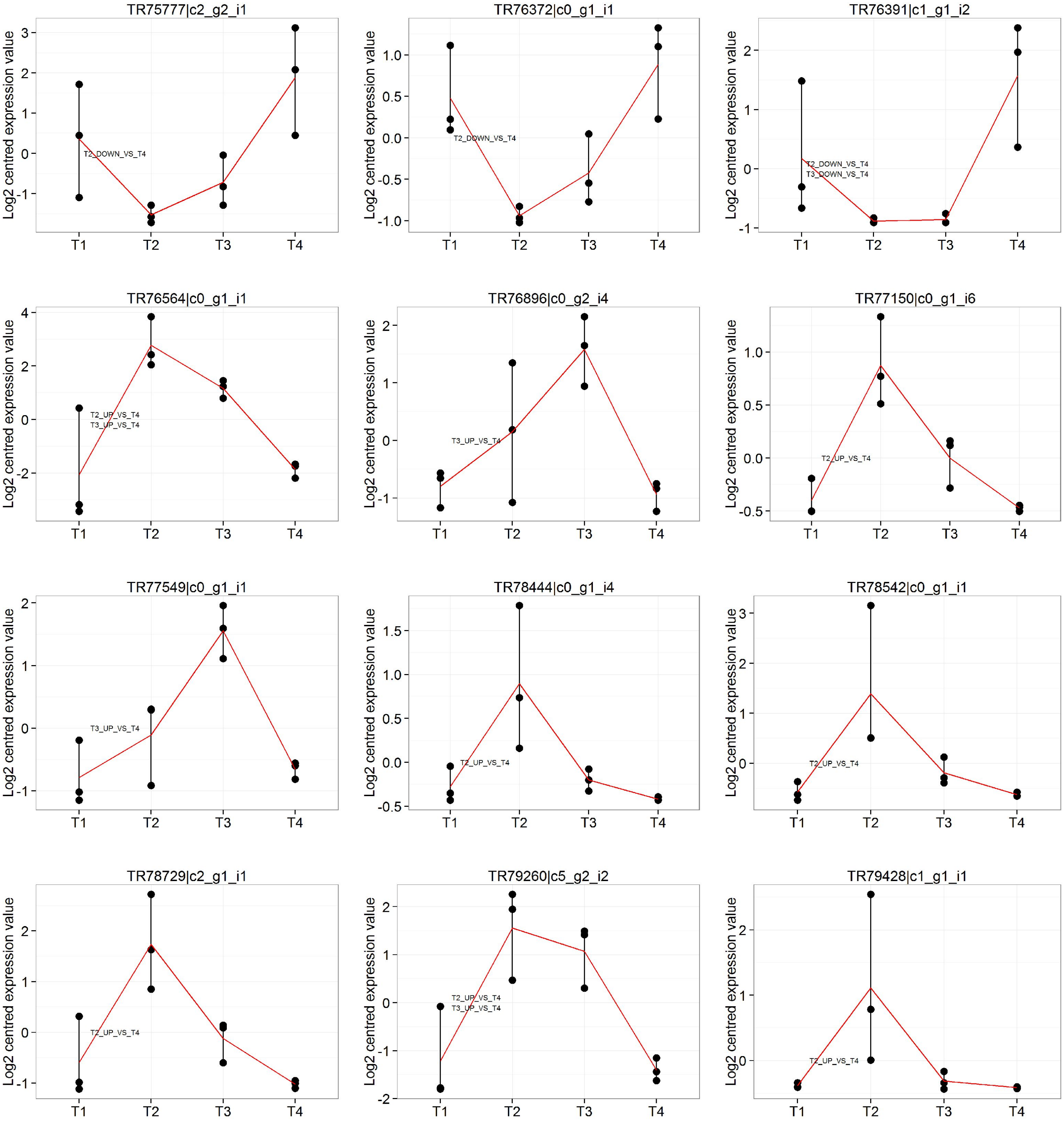
TR75777|c2_g2_i1
T2_DOWN_VS_T4
TR76372|c0_g1_i1
T2_DOWN_VS_T4
TR76391|c1_g1_i2
T2_DOWN_VS_T4
T3_DOWN_VS_T4
TR76564|c0_g1_i1
T2_UP_VS_T4
T3_UP_VS_T4
TR76896|c0_g2_i4
T3_UP_VS_T4
TR77150|c0_g1_i6
T2_UP_VS_T4
TR77549|c0_g1_i1
T3_UP_VS_T4
TR78444|c0_g1_i4
T2_UP_VS_T4
TR78542|c0_g1_i1
T2_UP_VS_T4
TR78729|c2_g1_i1
T2_UP_VS_T4
TR79260|c5_g2_i2
T2_UP_VS_T4
T3_UP_VS_T4
TR79428|c1_g1_i1
T2_UP_VS_T4
Log2 centred expression value
T1
T2
T3
T4

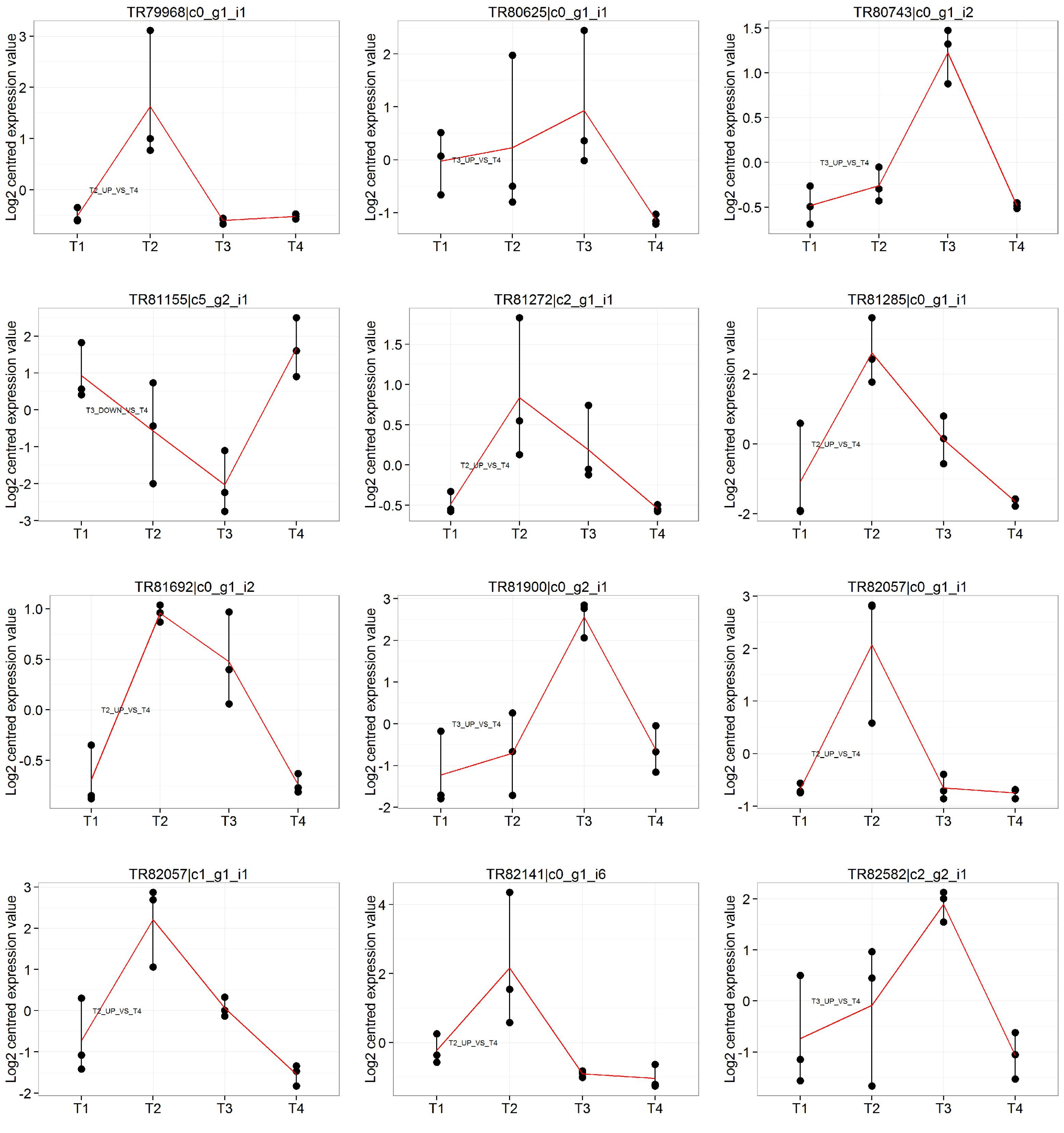
TR79968|c0_g1_i1
T2_UP_VS_T4
TR80625|c0_g1_i1
T3_UP_VS_T4
TR80743|c0_g1_i2
T3_UP_VS_T4
TR81155|c5_g2_i1
T3_DOWN_VS_T4
TR81272|c2_g1_i1
T2_UP_VS_T4
TR81285|c0_g1_i1
T2_UP_VS_T4
TR81692|c0_g1_i2
T2_UP_VS_T4
TR81900|c0_g2_i1
T3_UP_VS_T4
TR82057|c0_g1_i1
T2_UP_VS_T4
TR82057|c1_g1_i1
T2_UP_VS_T4
TR82141|c0_g1_i6
T2_UP_VS_T4
TR82582|c2_g2_i1
T3_UP_VS_T4
Log2 centred expression value
T1
T2
T3
T4

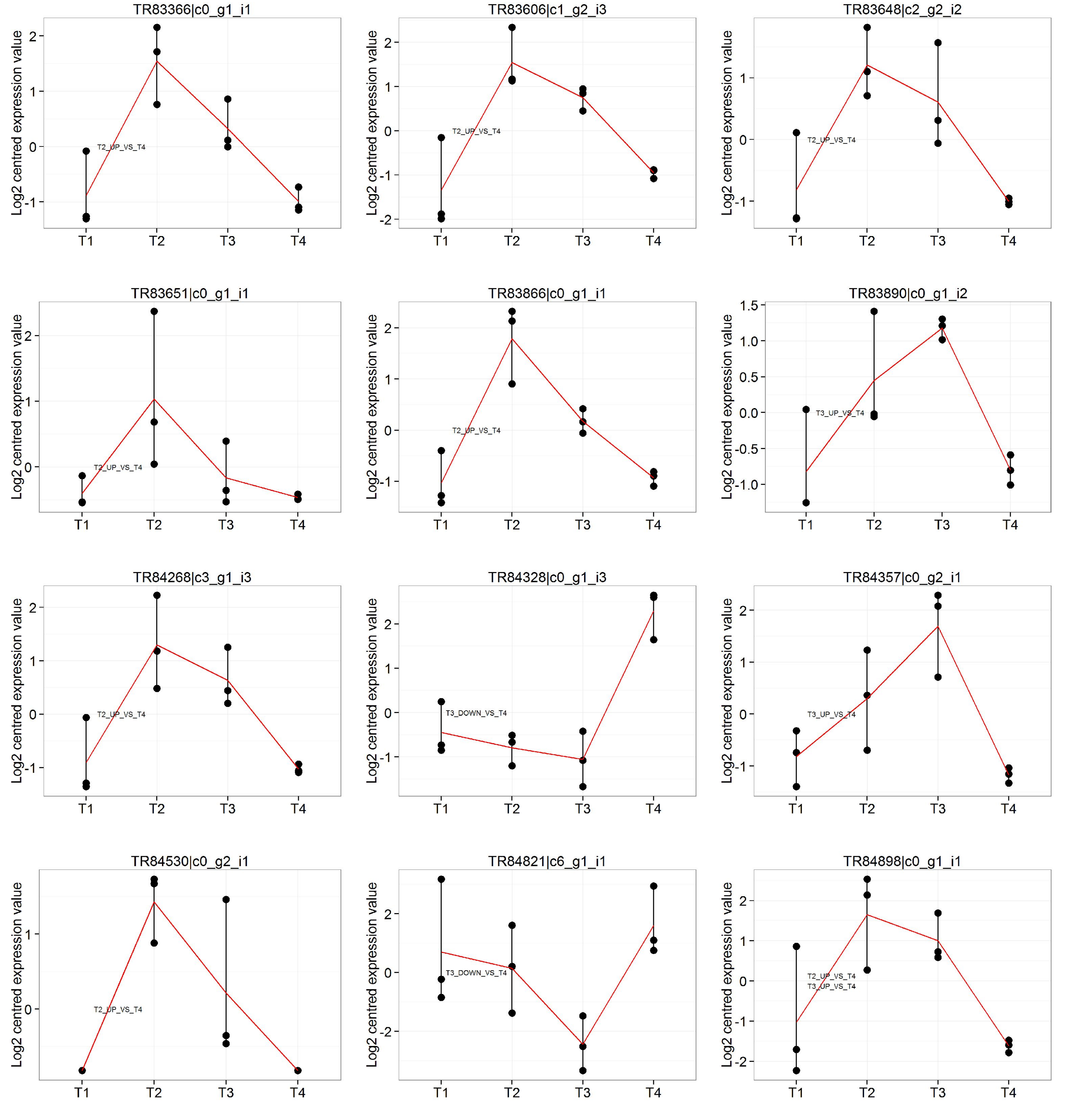
TR83366|c0_g1_i1
Log2 centred expression value
T2_UP_VS_T4
T1 T2 T3 T4
TR83606|c1_g2_i3
Log2 centred expression value
T2_UP_VS_T4
T1 T2 T3 T4
TR83648|c2_g2_i2
Log2 centred expression value
T2_UP_VS_T4
T1 T2 T3 T4
TR83651|c0_g1_i1
Log2 centred expression value
T2_UP_VS_T4
T1 T2 T3 T4
TR83866|c0_g1_i1
Log2 centred expression value
T2_UP_VS_T4
T1 T2 T3 T4
TR83890|c0_g1_i2
Log2 centred expression value
T3_UP_VS_T4
T1 T2 T3 T4
TR84268|c3_g1_i3
Log2 centred expression value
T2_UP_VS_T4
T1 T2 T3 T4
TR84328|c0_g1_i3
Log2 centred expression value
T3_DOWN_VS_T4
T1 T2 T3 T4
TR84357|c0_g2_i1
Log2 centred expression value
T3_UP_VS_T4
T1 T2 T3 T4
TR84530|c0_g2_i1
Log2 centred expression value
T2_UP_VS_T4
T1 T2 T3 T4
TR84821|c6_g1_i1
Log2 centred expression value
T3_DOWN_VS_T4
T1 T2 T3 T4
TR84898|c0_g1_i1
Log2 centred expression value
T2_UP_VS_T4
T3_UP_VS_T4
T1 T2 T3 T4

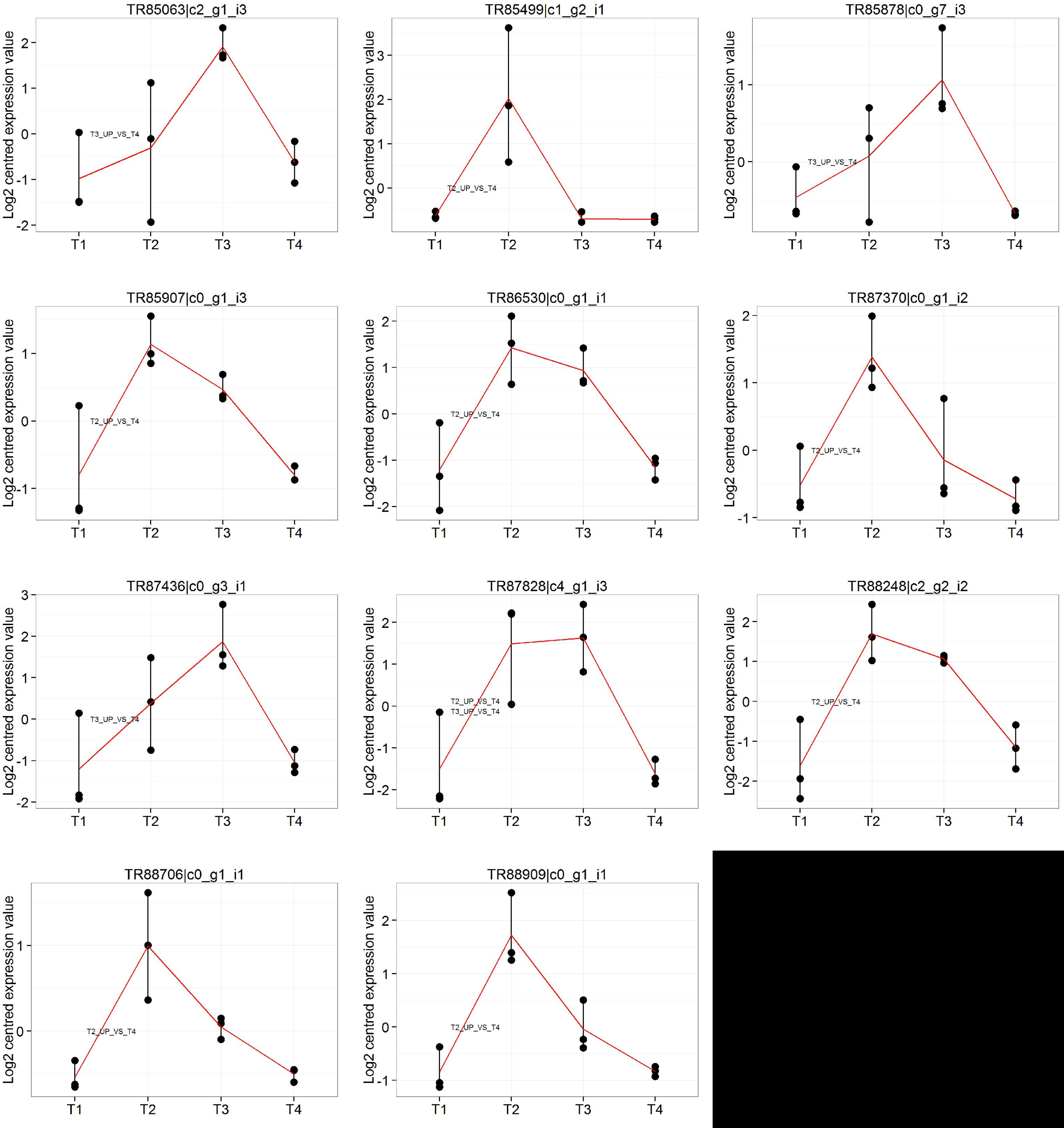
TR85063|c2_g1_i3
T3_UP_VS_T4
TR85499|c1_g2_i1
T2_UP_VS_T4
TR85878|c0_g7_i3
T3_UP_VS_T4
TR85907|c0_g1_i3
T2_UP_VS_T4
TR86530|c0_g1_i1
T2_UP_VS_T4
TR87370|c0_g1_i2
T2_UP_VS_T4
TR87436|c0_g3_i1
T3_UP_VS_T4
TR87828|c4_g1_i3
T2_UP_VS_T4
T3_UP_VS_T4
TR88248|c2_g2_i2
T2_UP_VS_T4
TR88706|c0_g1_i1
T2_UP_VS_T4
TR88909|c0_g1_i1
T2_UP_VS_T4
Log2 centred expression value
T1
T2
T3
T4

Supplementary table S1. General transcriptome assembly information and quality statistics for the five actinarian species used to identify conserved open reading frames (*Nemanthus annamensis*, *Telmatactis sp.*, *Anthopleura buddemeieri*, *Aulactinia veratra*, and *Actinia tenebrosa*) as well as a previous *Calliactis polypus* transcriptome used for comparison of transcript assembly.

**General details**					**Trinity stats (all transcript contigs)**			
Sample	Sampled tissues	After CD-HIT clustering? (95% identity)	SRA Accession	BUSCO summarised benchmark	No. Transcripts	%GC	N50 (bp)	Median contig (bp)
Nemanthus annamensis	All	Yes	SRR3228732	C:92%[D:24%], F:4.1%, M:3.6%, n:843	88325	40.49	1699	427
Telmatactis sp.	All	Yes	SRR3225580	C:58%[D:12%], F:26%, M:15%, n:843	131812	38.79	727	318
Anthopleura buddemeieri	All	Yes	SRR3205971	C:82%[D:24%], F:11%, M:6.0%, n:843	181029	40.83	861	383
Aulactinia veratra	All	Yes	SRR3205707, SRR3205708	C:89%[D:22%], F:5.8%, M:5.1%, n:843	144326	39.73	1090	388
Actinia tenebrosa	All	Yes	SRR3206038	C:91%[D:18%], F:3.7%, M:4.7%, n:843	105145	39.23	1609	387
Calliactis polypus (previous)	All	No	SRR3206038	C:92%[D:37%], F:3.9%, M:3.6%, n:843	214675	37.63	1577	356

Supplementary table S2. General transcriptome assembly information and quality statistics for all sampled specimens including the combined reference transcriptome used for differential gene expression analysis of *Calliactis polypus* after injury. Note that sample A1T2 received a poor BUSCO score due to ribosomal contamination.

**General details**			**Trinity stats (all transcript contigs)**			
Sample	Sampled tissues	BUSCO summarised benchmark	No. Transcripts	%GC	N50 (bp)	Median contig (bp)
A1T1	All	C:65%[D:17%], F:21%, M:12%, n:843	80850	38.96	1211	442
A1T2	All	C:26%[D:5.5%], F:27%, M:46%, n:843	63675	39.36	919	381
A1T3	All	C:63%[D:15%], F:23%, M:13%, n:843	73017	39.00	1258	448
A1T4	All	C:83%[D:27%], F:12%, M:4.5%, n:843	90571	38.70	1651	480
A2T1	All	C:74%[D:25%], F:16%, M:8.7%, n:843	94353	38.58	1339	450
A2T2	All	C:55%[D:16%], F:23%, M:20%, n:843	73673	39.16	1152	428
A2T3	All	C:75%[D:25%], F:14%, M:9.4%, n:843	99404	38.67	1442	441
A2T4	All	C:63%[D:19%], F:24%, M:12%, n:843	80236	36.55	1227	447
A3T1	All	C:51%[D:13%], F:27%, M:20%, n:843	74069	39.29	958	413
A3T2	All	C:72%[D:21%], F:17%, M:9.8%, n:843	80771	38.75	1523	469
A3T3	All	C:75%[D:20%], F:15%, M:9.4%, n:843	78468	38.88	1538	471
A3T4	All	C:70%[D:20%], F:18%, M:11%, n:843	79694	38.96	1210	443
Reference	All	C:91%[D:39%], F:4.6%, M:3.6%, n:843	252263	37.93	1167	385

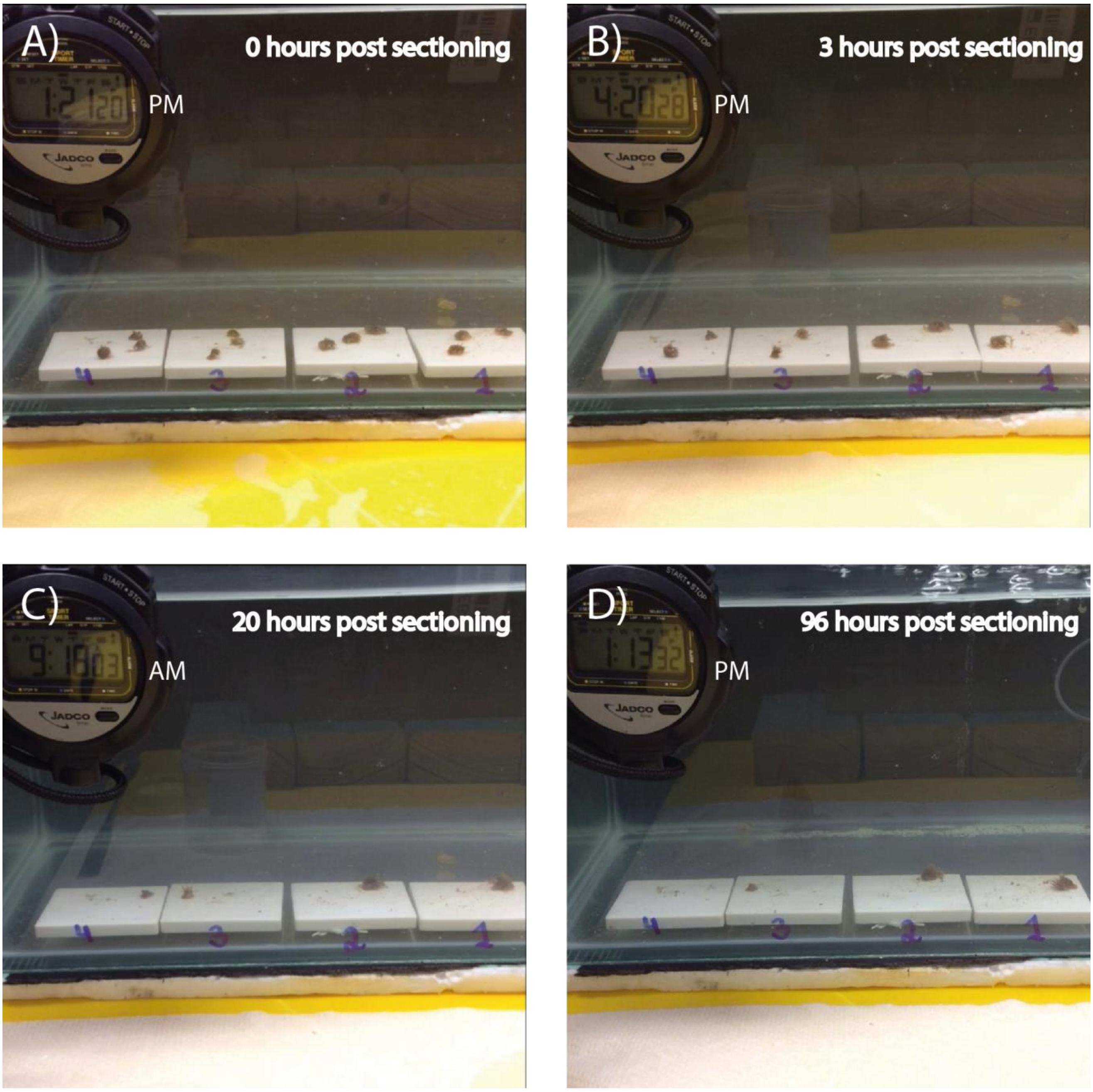

Supplementary Figure S1. Images extracted from a time-lapse video recording of *Calliactis polypus* following sectioning for our current study. Stopwatch in top left of each image depicts the time in 12-hour clock notation. Note that a fourth anemone is present in these images, although only three were used for our RNA-Seq analysis. A) Image taken shortly after initial *C. polypus* sectioning. The control (0 hps) anemone fragment was immediately removed and frozen in liquid Nitrogen. B) Image taken shortly after 3 hps sample was removed and frozen in liquid Nitrogen. C) Image taken shortly after 20 hps sample was removed and frozen in liquid Nitrogen. D) Image taken just before the final 96 hps sample

was removed and frozen in liquid Nitrogen. Note that the *C. polypus* fragment on tile ‘4’ is not in frame as it moved off the tile. hps; hours post sectioning.

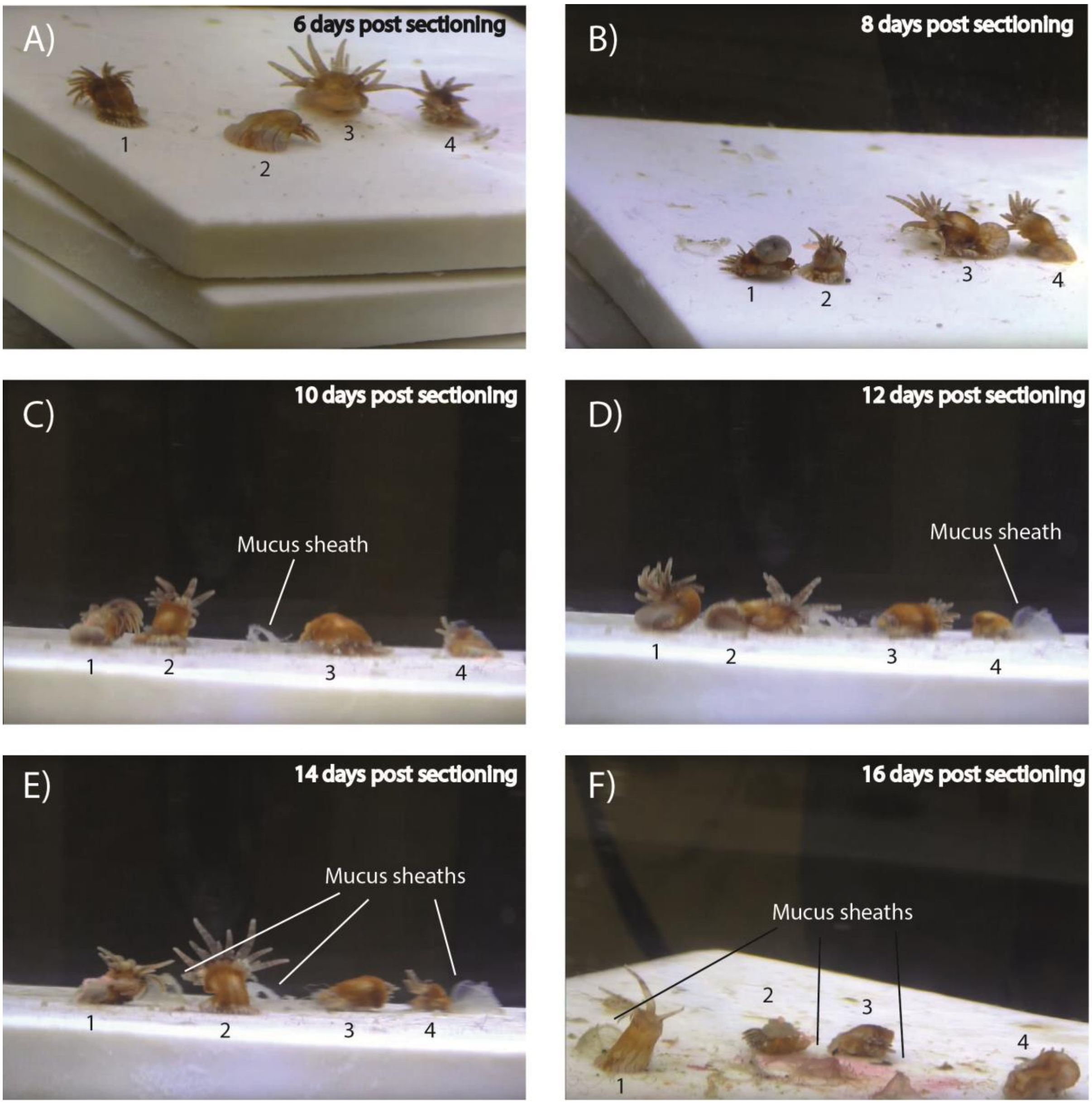

Supplementary Figure S2. Images extracted from a time-lapse video recording of *Calliactis polypus* following sectioning in a preliminary investigation of *C. polypus* regeneration (i.e., not from this study). Note that all sea anemone fragments survived beyond the timeframe indicated by these images. A) Image taken 6 dps with individual fragments indicated. No anemone fragments have as yet sutured their body into a radially symmetrical column, indicating that regeneration is still occurring. Fragment labelled ‘2’ demonstrates common

behaviour seen of ‘sheltering’ the opening in its body by flattening itself to the ground. B) Image taken 8 dps with individual fragments indicated. Fragment labelled ‘1’ at this time was highly mobile, using its tentacles to move across the tile. C) Image taken 10 dps with individual fragments indicated. Up until this point all anemones evidenced a thin, solidified layer of mucus covering the area that was sectioned. This mucus “sheath” was produced shortly after wounding. At this time, a mucus sheath can be seen detached from one of the anemone fragments. Additionally, although blurry, pink mesenterial filaments can be seen in the fragment labelled ‘1’ which was seen in other fragments throughout the experiment. D) Image taken 12 dps with individual fragments indicated. A mucus sheath can be seen detached from one of the anemone fragments. Fragment labelled ‘1’ is demonstrating a common behaviour of spasmodic convulsions involving the folding in and flexing out of its body which other fragments occasionally performed. E) Image taken 14 dps with individual fragments indicated. Multiple mucus sheaths can be seen detached from the anemone fragments. F) Image taken 16 dps with individual fragments indicated. Multiple mucus sheaths can be seen detached from the anemone fragments. Fragment labelled ‘1’ has fully sutured its column back into a radially symmetric form, indicating that regeneration may be complete in this fragment. Fragment labelled ‘2’ has still not achieved this, although fragments ‘3’ and ‘4’ have almost sutured their columns back into a radially symmetric form. Pink staining is thought to be the result of pink mesenterial filament decomposition. dps; days post sectioning.
